# Health literacy among lactating women and the promotion of breastfeeding and maternal and child health

**DOI:** 10.1590/1980-220X-REEUSP-2025-0180en

**Published:** 2025-10-03

**Authors:** Élida Cristina Santos Corrêa, Poliana Pereira Costa Rabelo, Pablo Nascimento Cruz, Isaura Letícia Tavares Palmeira Rolim, Katarinne Lima Moraes, Janaína Miranda Bezerra

**Affiliations:** 1 Universidade Federal do Maranhão, Programa de Pós-Graduação em Enfermagem, São Luís, MA, Brazil.; 2 Universidade Brasília Ceilândia, Faculdade de Ciências e Tecnologias em Saúde, Brasília, DF, Brazil.; 3 Universidade Federal do Maranhão, Campus Imperatriz, MA, Brazil.

**Keywords:** Health literacy, Breast Feeding, Nursing

## Abstract

**Objective:**

To describe and analyze the functional health literacy of lactating women treated at a Human Milk Bank in Maranhão.

**Method:**

Cross-sectional, descriptive study, carried out between August 2023 and March 2024, at the Milk Bank of a public university hospital in Northeast Brazil, using a validated health literacy scale.

**Results:**

A total of 187 lactating women participated, of which 56.15% had inadequate health literacy. There was a significant association between health literacy and the Mini Mental State Examination, with a greater probability of inadequate literacy among younger women (67.1%), with finished elementary education (91.7%), unfinished secondary education (91.7%), and income below one minimum wage (95%). Association tests (Chi-square) were applied, with significance at p < 0.05, highlighting the association between literacy and age (p = 0.00697).

**Conclusion:**

Most of the lactating women evaluated presented inadequate health literacy, showing the importance of educational strategies as a basis for health promotion and improvement of the care provided to this population.

## INTRODUCTION

Health Literacy (HL) is associated with personal understanding, encouragement, and the ability to make decisions regarding health throughout life. Some of these skills include numeracy, reading, writing, and the ability to express views, communicate effectively, and use technology. In this context, HL has become essential for health promotion and improvement in health decision-making^(1)^.

In contrast, when this level is low, the impacts can be negative, as in the case of Breastfeeding (BF). Mothers with low HL tend to have less understanding and access to information about breastfeeding, which can hinder its continuity. Therefore, knowledge about the benefits of breastfeeding, for both mother and baby, is essential for the success of this practice^(2)^.

International studies, such as that by Valero-Chillerón et al.^(3)^, held in Valencia, Spain, and by Fatmawati et al.^(4)^, in Malang, Indonesia, analyzed the influence of health literacy on exclusive breastfeeding up to six months of age. Furthermore, research carried out in Spanish hospitals focused on creating specific instruments to assess breastfeeding literacy. These studies reinforce the need for further research in Brazil on the impact of health literacy on breastfeeding practices, considering the country’s cultural and social context^(3,4)^.

The SAHLPA-18 instrument has been validated and applied in different contexts, as in the study by Campos et al.^(5)^, which investigated the association of functional health literacy among women served by the Family Health Strategy, in the state of São Paulo, and in the study by Lima et al.^(6)^, which analyzed the level of functional literacy in primary care users in the state of Pará.

More recent data shows that the global rate of Exclusive Breastfeeding (EBF) up to 6 months reached 48%, approaching the target of 50% for the year 2025. However, there are regional disparities: Rwanda reached 87%, Sri Lanka 82%, Pakistan 37%, and the United States 25%. Factors such as breastfeeding support policies and education affect these rates, as do short maternity leaves^(7,8)^.

In Brazil, EBF rates have been increasing over the years, highlighting efforts in public policies aimed at promoting, protecting, and supporting breastfeeding. Programs such as the Baby-Friendly Hospital Initiative (*IHAC*) and the *Estratégia Amamenta e Alimenta Brasil* (Breastfeed and Feed Brazil Strategy), combined with increased access to information and the creation of human milk banks, have contributed to this progress. According to the National Study of Child Feeding and Nutrition (ENANI), in 2020 a rate of 45.7% of EBF was found in children under six months of age^(9)^.

In the state of Maranhão, according to the National Food and Nutrition Surveillance System (*SISVAN*), data from 2021 show that the EBF rate in children aged 0 to 6 months was 47%, while the percentage of Continued Breastfeeding (CBF) reached 53%^(10)^.

Given the fragility of the EBF scenario in the State of Maranhão, this research emphasizes the importance of health education as a way to encourage BF at all levels of care. In this sense, identifying the level of health literacy of women treated at the Human Milk Bank (HMB) of a public university hospital in the state of Maranhão contributes to future interventions that promote both EBF and CBF. The observation of the lack of data on this topic in Brazil, especially in the Northeast region^(5)^, motivated the formulation of the following research question: does Health Literacy contribute to the promotion of Exclusive Breastfeeding?

Although this cross-sectional study identifies significant associations between functional health literacy and variables related to breastfeeding, it is not possible to assert causality between the factors. Nevertheless, the findings suggest relevant avenues for future research and public health interventions.

HL plays an important role in improving health services and achieving positive outcomes for the lives of modern citizens. This is an essential combination of social capital that must be seen as an integrated policy, not only in the health sector, but also in other sectors^(2)^. HL is essential for the advancement of public policies at local, national and global levels, promoting improved knowledge about breastfeeding among lactating women^(6)^.

In this context, the study aimed to describe and analyze the functional health literacy of lactating women treated at a human milk bank in the state of Maranhão.

## METHOD

### Design of Study

This is a cross-sectional, descriptive study.

Cross-sectional studies, in particular, are used to measure the prevalence of characteristics, behaviors and health conditions in a given population. Because data are collected only once, this type of study is useful for understanding the distribution of a situation in a sample and identifying associations between variables. However, it does not allow determining cause and effect relationships, since there is no monitoring over time^(11)^.

The quantitative method focuses on the study of measurable levels of reality, with the aim of identifying and interpreting observable and analyzable data, indicators and trends in an objective manner^(12)^.

### Study Local

The research setting was the Human Milk Bank of the public university hospital in the Northeast region of Brazil, state of Maranhão. The institution is certified as a teaching hospital and recognized as a Baby-Friendly Hospital, integrating the National Network of Human Milk Banks and following the guidelines of the Brazilian Health Regulatory Agency (Anvisa). The HMB has a multidisciplinary team and adequate structure to assist lactating women, including offices, a collection room, a lecture room, space for processing and pasteurizing human milk, as well as bedside care in the Rooming-in. The service provides daily care, both by spontaneous demand and through appointment, with a monthly average of 38 nursing mothers served and approximately 420 consultations in total.

### Population and Selection Criteria

A total of 187 lactating women treated at the aforementioned hospital unit participated in this study. They voluntarily agreed to participate in answering the study questionnaires. The following inclusion criteria were considered to define the participants: being a nursing mother aged 18 years or older, being attended by the Human Milk Bank team at the public university hospital in the Northeast region of Brazil in the Collection Room or directly at the Human Milk Bank, breastfeeding babies up to 6 months of age, and having cognitive and communicative capacity, according to the Mini Mental State Examination (MMSE), to understand and answer questions related to health literacy and breastfeeding. During participants recruitment, 15 lactating women were excluded for presenting results below the recommended scores in the MMSE, with a normal score being ≥ 25 points, mild cognitive loss ranging from 21 to 24 points, moderate cognitive loss from 10 to 20 points, and severe cognitive loss being ≤ 9 points. Only lactating women with normal scores or mild cognitive impairment were included in the study, resulting in 187 eligible lactating women.

### Sample Size Calculation

The sample size was calculated considering an estimated proportion of 50% for the main outcome, a 95% confidence level (Z = 1.96), and a margin of error of 5% (0.05), which resulted in a minimum required sample of 187 lactating women. Considering an average of 30 monthly visits to the health unit, sampling was carried out randomly based on consultations with the medical records of the patients treated. In total, 202 women were interviewed. However, 15 participants were excluded for not meeting the inclusion criteria, totaling a final sample of 187 women, a number that exceeds the minimum necessary defined by the sample calculation, providing greater robustness and reliability to the results obtained.

### Data Collection

Data were collected between August 2023 and March 2024 at the Human Milk Bank of the university hospital in the state of Maranhão. The team responsible for administering the instruments was composed of the main researcher, an undergraduate student, and a master’s student, who received prior training to standardize the application, clarify doubts, and guarantee ethical aspects, ensuring data quality.

Collection took place from Monday to Friday, from 8 am to 5 pm, HMB opening hours. Lactating women were randomly approached in the waiting room, where the research objectives were explained. Participants who agreed signed the Free and Informed Consent Form (FICF) before instruments application.

This moment occurred in three parts. First, with the collection of sociodemographic data through a semi-structured questionnaire developed based on the study by Suárez-Cotelo^(13)^. The following demographic characteristics were considered: age, marital status, level of education, monthly income, and municipality of residence. Second, the Mini Mental State Examination (MMSE) instrument was applied^(14)^ to assess mental and cognitive status. As a research instrument, it has been used in population epidemiological studies, integrating several neuropsychological batteries^(15)^. The instrument considers the following cognitive classification: normal, ≥ 25 points; mild cognitive loss ranges from 21 to 24 points; moderate cognitive loss, from 10 to 20 points; and severe cognitive loss, ≤ 9 points. Third, the Short Assessment of Health Literacy for Portuguese-Speaking Adults, SAHLPA-18^(16)^, was administered, an instrument that aims to assess the literacy of individuals in health environments, classifying it as inadequate (score between 0-14) or adequate (score > 15).

### Data Analysis and Treatment

Data were tabulated in Microsoft Excel 2021 and analyzed in RStudio (version 2023.12.1). To verify the association between the variables, the Chi-square test was used, considering health literacy and the Mini Mental State Examination as dependent variables, and age as the independent variable, adopting a significance level of p < 0.05. Due to the presence of categories with an expected frequency of less than five, the associations between education, income and health literacy were assessed using Fisher’s exact test. The proportions were accompanied by their respective 95% confidence intervals, calculated using Wilson’s exact method. The results were organized in tables and discussed in light of the scientific literature relevant to the topic.

### Ethical Aspects

The study addressed ethical issues related to human beings, in accordance with Resolution No. 466/12 and Resolution No. 510/16 of the National Health Council. This study is an integral part of the umbrella project “Suficiência do leite materno: conhecimento de nutrizes” (“Breast milk sufficiency: nursing mothers knowledge”), approved by the Scientific Committee of the University Hospital of UFMA and by the Research Ethics Committee of the Federal University of Maranhão, via Plataforma Brasil, with Opinion No. 5,310,907. All participants received guidance and clarification about the research, as well as the FICF, which was duly signed after participants agreeing to participate in the research.

The writing of this study followed the recommendations of the STROBE guideline for observational studies.

## RESULTS

A total of 187 lactating women were included in the analysis. The group studied was characterized according to sociodemographic and health conditions (Tables 1 and 2). Regarding age, the majority of participants, 45.46% (85), were between 18 and 26 years old. Regarding marital status, 40.64% (76) of lactating women were single. As per education, 54.54% (102) had finished high school, and 78.07% (146) survived on a monthly income of between 1 and 3 minimum wages, noting that the minimum wage in effect at the time of collection was R$ 1,320.00. Geographically, 86% (161) of lactating women resided in São Luís.

**Table 1:** Description of sociodemographic data of lactating women treated at the human milk bank of the public university hospital, São Luís – MA, Brazil, 2024.

Variable	Frequency	
	n	%
Age (years)		
18 to 26	85	45.46
27 to 35	78	41.71
36 to 44	24	12.83
Marital status		
Single	76	40.64
Common-law marriage	55	29.41
Married	50	26.74
Divorced	5	2.68
Widow	1	0.53
Level of education		
Unfinished elementary school	2	1.07
Finished elementary school	12	6.42
Unfinished high school	21	11.23
Finished high school	102	54.54
Unfinished higher degree	16	8.56
Finished higher degree	30	16.04
Graduate studies	4	2.14
Income		
Less than 1 minimum wage	20	10.7
From 1 to 3 minimum wages	146	78.07
From 4 to 6 minimum wages	18	9.63
Greater than 7 minimum wages	3	1.6
Municipality of residence		
São Luís	161	86
Others	23	14
**TOTAL**	**187**	**100**

**Note:** The minimum wage in effect during the collection period (Aug/2023 to Mar/2024) was R$1,320.00.

Source: Prepared by the author (2024).

**Table 2:** Description of information about previous breastfeeding of lactating women treated at the human milk bank of the public university hospital, São Luís – MA, Brazil, 2024.

Variables	Frequency	
	n	%
Did you receive guidance on breastfeeding?		
Never received guidance	18	9.63
Prenatally and after birth	69	36.9
Only after birth	98	52.4
Only in prenatal care	2	1.07
Previous experience with breastfeeding?		
No, this is her first child	98	52.4
Yes, she has breastfed more than one baby.	14	7.49
Yes, she has already breastfed another baby.	75	40.11
If have already breastfed, how long?		
Never breastfed before	98	52.41
< 6 months	12	6.42
6 months	3	1.6
> 6 months and < 2 years	48	25.67
> 2 years	26	13.9
**TOTAL**	**187**	**100**

Source: Prepared by the author (2024).

In Table 2, there is a significant variation in the obtainment of guidance on breastfeeding, with 52.4% (98) of women receiving some guidance on breastfeeding only after giving birth. Regarding breastfeeding experience, 52.4% (98) of the women were primiparous. Among those who had already breastfed, the majority, 25.67% (48), continued breastfeeding for a period longer than 6 months, but less than 2 years.

The assessment of the health literacy level of lactating women was carried out using the SAHLPA-18, frequently used in health care settings. The research results indicated that 56.15% (105) of the participants had inadequate literacy, while 43.85% (82) demonstrated adequate literacy.

When analyzing the association between cognitive function, assessed by the Mini Mental State Examination (MMSE), and the level of health literacy (HL), a statistically significant association was identified (Chi-square test = 9.13; p = 0.0025). Among women with normal cognitive function, 51.0% (75) had inadequate literacy (95% CI: 43.0–58.9%), while 49.0% (153) had adequate literacy (95% CI: 41.1–57.0%). Among those with mild cognitive loss, 79.4% (7) had inadequate literacy (95% CI: 63.2–90.2%) and only 20.6% (34) had adequate literacy (95% CI: 9.8–36.8%). These findings indicate that women with lower cognitive performance have a higher frequency of inadequate health literacy, compared to those with preserved cognitive function.

When analyzing the association between age group and health literacy, a statistically significant difference was identified (Chi-square test = 9.93; p = 0.00697). It was observed that inadequate literacy was more prevalent among younger women: in the age group of 18 to 26 years, 67.1% (57) presented inadequate literacy (95% CI: 56.3%–76.4%); between 27 and 35 years, 51.3% (40) (95% CI: 39.8%–62.7%); and between 36 and 44 years, the frequency was 33.3% (8) (95% CI: 17.3%–52.8%). These findings suggest that health literacy tends to increase with age.

Among women with finished primary education, 91.7% (11 out of 12) had inadequate literacy (95% CI: 61.5%–99.8%). In contrast, among those with complete higher education, only 16.7% (5 in 30) presented inadequate literacy (95% CI: 5.6%–36.7%), indicating a significant association between education level and health literacy level (Fisher’s exact test, p < 0.001).

Among women with income below 1 minimum wage, 95.0% (19 out of 20) had inadequate literacy (95% CI: 75.1%–99.9%). Among those with an income of 4 to 6 minimum wages, 33.3% (6 out of 18) presented inadequate literacy (95% CI: 13.3%–59.0%). The association between family income and health literacy level was statistically significant (Fisher’s exact test, p = 0.0007).

## DISCUSSION

Sociodemographic characteristics directly influence the level of HL among lactating women, an aspect that proved to be relevant for understanding the profile of this group. The study revealed a predominance of young adult women among the lactating women evaluated, the majority of whom are single and have finished high school. It is worth highlighting that, although HL is interdependent on education, even in this sample with a predominance of lactating women with high school education, HL was inadequate. Most have a family income between 1 and 3 minimum wages. These data emphasize the importance of adapting health education actions to the specific needs of this population. Furthermore, HL is influenced by more complex social structures than those described by educational level alone^(5)^.

These findings are in line with other research carried out in Brazil, such as the study in Altamira-PA, which analyzed the FHL of Primary Health Care users, identifying age, education and income as predictive factors of the population’s FHL^(6)^. Another study, focused on breast cancer prevention, also showed that variables such as age, income, and education significantly influence the adoption of preventive practices and healthy habits among women^(17)^.

These data indicate that sociodemographic factors, such as age group, level of education and economic context, directly affect the lifestyle of breastfeeding women and, consequently, the well-being of the mother-baby binomial. Therefore, when planning health education actions, it is essential that interventions are adapted to the specificities of each group. This approach can improve the impact of health education strategies, promoting more effective and sustainable care for mothers and babies^(18)^.

Regarding the previous breastfeeding experience of lactating women, it is clear that most women only received guidance after giving birth. This data reveals a deficit in preparation during the prenatal period, highlighting the need to improve interventions aimed at breastfeeding. Ensuring that breastfeeding women receive guidance both before and after birth is essential to promote appropriate practices from the beginning of breastfeeding, enabling knowledge of the benefits of EBF and preventing the consequences of not providing it until the baby’s sixth month of life^(19)^.

A study conducted in Imperatriz, Maranhão, found that 43.9% of women received guidance on breastfeeding only in the maternity ward, highlighting the impact of obstetric history on breastfeeding self-efficacy and the need to reinforce counseling during prenatal care^(20)^. This study is corroborated by an international study, which demonstrated that educational interventions both prenatally and postpartum are effective in increasing self-confidence and effectiveness in breastfeeding. These data reinforce the importance of comprehensive and educational prenatal care, which offers the necessary support for lactating women to initiate breastfeeding safely and effectively^(21)^.

Based on the data obtained in this study, the majority of women analyzed had no previous experience with breastfeeding, largely because they were primiparous. The literature indicates that breastfeeding success is often associated with the number of pregnancies and births, as studies indicate that multiparous mothers tend to have greater self-confidence and effectiveness in breastfeeding^(20)^.

With the application of SAHLPA-18, lactating women who presented inadequate HL stood out in this research. The results obtained corroborate another study carried out at the HMB of the public university hospital in the Northeast region of Brazil, in which the assessment instrument was the Health Literacy Scale (HLS-14), and 52% of the puerperal women also presented inadequate HL^(22)^. Although both studies reveal a high rate of inadequate HL, the main difference lies in the instrument used: the SAHLPA-18, which focuses on the understanding of medical terms, and the HLS-14, which assesses the understanding of health information in different contexts.

Research conducted at Kosar Hospital in Qazvin, Iran, found that the HL of lactating women was generally satisfactory, while another study, from the same country, conducted in comprehensive health care centers in the city of Rafsanjan, indicated that 71.8% of mothers had adequate to excellent HL^(23)^. In contrast, other findings in the same country showed that most lactating women had low HL^(24)^.

Cognitive function and HL are essential elements for the well-being of lactating women, directly influencing their ability to understand and apply health information during pregnancy and the postpartum period. The results of this study corroborate the findings of a study that analyzed the level of HL and cognitive performance in a group of pregnant and postpartum women. This study revealed that 41.6% of women had difficulty understanding and dealing with health-related information, especially those with less education, younger women, and non-white women. Furthermore, it was observed that postpartum women were more likely to have reduced performance in aspects such as reasoning speed and problem-solving. These findings highlight the importance of considering both social and biological factors when developing strategies to assess health understanding and cognition in vulnerable groups^(25)^.

The association between the MMSE result and HL demonstrates that, even with a subtle cognitive decline, there is a tendency for HL to decrease. This relationship is particularly noticeable in older adults, where reduced functions such as memory and mental flexibility may predict a reduced ability to understand and manage health-related information^(26)^.

The HL level showed lower performance among lactating women aged 18 to 26 years. This association was reinforced by a study conducted at Sivas Numune Hospital in Türkiye, which showed the limitations of HL, especially among “first-time mothers”^(27)^. Despite having access to a wealth of health information, many of these mothers reported feeling overwhelmed and frightened by the sheer volume of information, which can hinder informed decision-making. Age, therefore, is a relevant factor influencing HL of lactating women, with primiparous and younger mothers facing significant challenges in understanding and applying health information effectively.

A significant relationship was observed between maternal age and HL, with higher scores for mothers aged between 36 and 44 years, which is in agreement with a study that also identified higher HL scores in older lactating women. This effect may be related to the greater experience of mothers in this age group. In general, higher maternal age is associated with higher HL scores, which in turn tends to favor a greater duration and likelihood of breastfeeding^(2,28,29)^.

Based on the data obtained in this study, it is observed that the level of education plays a crucial role in lactating women’s HL. The association between adequate HL and higher levels of education highlights the importance of a regular and successful educational trajectory, as also noted by the aforementioned author^(30)^.

Women with a higher level of education demonstrate greater commitment and ability to breastfeed assertively and for longer periods of time. This behavior can be attributed to the fact that these women have greater access to information about the benefits that EBF provides both to the mother and the child. Furthermore, it is expected that this factor will also contribute to better breast care during pregnancy and the postpartum period. In contrast, there is a tendency for breastfeeding women with less education to face more difficulties in the initial stages of the breastfeeding process, due to less access to information and preventive practices^(31)^.

Conversely, a study carried out with women served by the Family Health Strategy in the Southeast region of Brazil revealed that the indicator “complete years of study” may not reflect the level of literacy. This indicates that educational level alone does not guarantee adequate HL, since even highly educated users may have difficulty understanding terminology and procedures specific to the healthcare context^(5)^.

International literature indicates that lower levels of HL are more prevalent in low-income groups. A study conducted in Hawaii, USA, found that vulnerable mothers, with inadequate HL and low income, tend to make less favorable health decisions, which can negatively impact their own health care and the well-being of their children^(32)^.

Another study, carried out in Laos, a Southeast Asian country, found that HL is inadequate in both urban and rural areas of the country. Moreover, it highlighted family income as the main factor influencing this health outcome^(33)^.

Low-income women are more susceptible to low levels of HL, reinforcing the association between HL and family income. Income, as an indicator of social status, is directly linked to access to information and health services, negatively influencing HL levels in economically disadvantaged populations^(5)^.

Limitations of the study include its cross-sectional nature, the use of a non-probabilistic sample, the focus on a single municipality in Northeastern Brazil, and the absence of a qualitative analysis that considers cultural aspects of breastfeeding practices. Added to this is the absence of a multicenter approach at the regional or state level and the non-inclusion of other human milk banks in the region. Additionally, the group of participating breastfeeding women does not represent the entire female population, and a scarcity of scientific literature on health literacy in this specific population was identified in the Brazilian context. These factors reinforce the need for further research to broaden understanding of the health literacy of lactating women in different human milk banks across different regions of the country.

## CONCLUSION

It is concluded that the majority of lactating women treated at the Human Milk Bank in the state of Maranhão have inadequate functional health literacy. Data demonstrate a significant association between the level of HL and factors such as age, education, family income, and MMSE results. Younger lactating women, with less education, lower income, and signs of mild cognitive impairment had a higher prevalence of inadequate HL.

Given the findings of this study, it is highlighted that health literacy, as a conceptual basis for health promotion, is an effective health care strategy for breastfeeding women. Its adoption as a guideline in public policies can favor empowerment, self-care, and expanded access to qualified health services, contributing to improved outcomes related to breastfeeding and maternal and child health.

Although the prevalence of inadequate HL in this study is high, it is suggested that resources be introduced to improve communication between health professionals and patients, especially in the case of lactating women with low income and education. Educational measures are essential for professionals to adapt their language, adjust guidelines and prescriptions, and develop communication tools that meet the needs and abilities of lactating women at greater risk of HL deficits.

It is suggested that health literacy be included in the ongoing training and qualification of primary care professionals, aiming to improve care for breastfeeding women. In healthcare practice, it is essential to adopt communication strategies appropriate to the sociocultural profile of users. Future research, preferably multicenter and qualitative, could broaden understanding of the topic in different regional contexts.

## DATA AVAILABILITY

All datasets supporting the findings of this study are published in the article itself.
