# Supplementary material for: Health literacy among lactating women and the promotion of breastfeeding and maternal and child health
Source: Rev Esc Enferm USP. 2025 Oct 3;59:e20250180. doi: 10.1590/1980-220X-REEUSP-2025-0180en (PMC12503122; doi:10.1590/1980-220X-REEUSP-2025-0180en)
Supplement: Translation

# Translation: Letramento em saúde entre mulheres lactantes e promoção do aleitamento materno e da saúde materno-infantil

**DOI:** 10.1590/1980-220X-REEUSP-2025-0180pt

**Authors:** Élida Cristina Santos Corrêa, Poliana Pereira Costa Rabelo, Pablo Nascimento Cruz, Isaura Letícia Tavares Palmeira Rolim, Katarinne Lima Moraes, Janaína Miranda Bezerra

**Affiliations:** 1 Universidade Federal do Maranhão, Programa de Pós-Graduação em Enfermagem, São Luís, MA, Brasil.; 2 Universidade Brasília Ceilândia, Faculdade de Ciências e Tecnologias em Saúde, Brasília, DF, Brasil.; 3 Universidade Federal do Maranhão, Campus Imperatriz, MA, Brasil.

**Keywords:** Letramento em saúde, Aleitamento materno, Enfermagem

## Abstract

**Objetivo:**

Descrever e analisar o letramento funcional em saúde de mulheres lactantes atendidas em um Banco de Leite Humano do Maranhão.

**Método:**

Estudo transversal, descritivo, realizado entre agosto/2023 e março/2024, no Banco de Leite de um hospital universitário público no Nordeste do Brasil, utilizando escala validada de letramento em saúde.

**Resultados:**

Participaram 187 mulheres lactantes, das quais 56,15% apresentaram letramento em saúde inadequado. Houve associação significativa entre letramento em saúde e o Mini Exame do Estado Mental, com maior probabilidade de letramento inadequado entre mulheres mais jovens (67,1%), com ensino fundamental completo (91,7%), médio incompleto (91,7%) e renda inferior a um salário mínimo (95%). Foram aplicados testes de associação (Qui-quadrado), com significância em p < 0,05, destacando-se associação entre letramento e idade (p = 0,00697).

**Conclusão:**

A maioria das lactantes avaliadas apresentou letramento em saúde inadequado, evidenciando a importância de estratégias educativas como base para a promoção da saúde e qualificação do cuidado prestado a esse público.

## INTRODUÇÃO

O Letramento em Saúde (LS) está associado ao entendimento pessoal, ao incentivo e à capacidade de tomar decisões em relação à saúde ao longo da vida. Algumas dessas capacidades incluem o conhecimento em números, leitura, escrita e a competência de externar pontos de vista, comunicar-se de forma eficaz e utilizar tecnologias. Nesse contexto, o LS tem se tornado essencial para a promoção da saúde e o aperfeiçoamento na tomada de decisões em saúde^(1)^.

Por outro lado, quando esse nível é baixo, os impactos podem ser negativos, como no caso do Aleitamento Materno (AM). Mães com baixo LS tendem a ter menor compreensão e acesso a informações sobre amamentação, o que pode prejudicar a continuidade do AM. Assim, o conhecimento sobre os benefícios da amamentação, tanto para a mãe quanto para o bebê, é essencial para o sucesso dessa prática^(2)^.

Estudos internacionais, como o de Valero-Chillerón et al.^(3)^, realizado em Valência, na Espanha, e de Fatmawati et al.^(4)^, em Malang, na Indonésia, analisaram a influência do letramento em saúde na amamentação exclusiva até os seis meses de vida. Além disso, pesquisas desenvolvidas em hospitais espanhóis concentraram-se na criação de instrumentos específicos para avaliar o letramento voltado à amamentação. Esses estudos reforçam a necessidade de mais investigações no Brasil sobre o impacto do letramento em saúde na prática da amamentação, considerando o contexto cultural e social do país^(3,4)^.

O instrumento SAHLPA-18 tem sido validado e aplicado em contextos distintos, como no estudo de Campos et al.^(5)^, que investigou a associação do letramento funcional em saúde (LFS) entre mulheres atendidas pela Estratégia Saúde da Família, no estado de São Paulo, e no estudo de Lima et al.^(6)^, que analisou o nível de letramento funcional em usuário da atenção primária no estado do Pará.

Dados mais recentes, mostram que a taxa global de Aleitamento Materno Exclusivo (AME) até os 6 meses chegou a 48%, aproximando-se da meta de 50% para o ano de 2025. No entanto, há disparidades regionais: Ruanda alcançou 87%, Sri Lanka, 82%, Paquistão, 37%, e os Estados Unidos, 25%. Fatores como políticas de apoio à amamentação e educação interferem nessas taxas, assim como licenças-maternidade curtas^(7,8)^.

No Brasil, as taxas de AME vêm aumentando ao longo dos anos, destacando os esforços em políticas públicas voltadas para promoção, proteção e apoio à amamentação. Programas como a Iniciativa Hospital Amigo da Criança (IHAC) e a Estratégia Amamenta e Alimenta Brasil, aliados à ampliação do acesso à informação e à criação de bancos de leite humano, têm contribuído para esse avanço. Conforme o Estudo Nacional de Alimentação e Nutrição Infantil (ENANI), em 2020 foi constatado um índice de 45,7% de AME em crianças menores de seis meses de idade^(9)^.

No estado do Maranhão, conforme o Sistema Nacional de Vigilância Alimentar e Nutricional (SISVAN), dados de 2021 mostram que a taxa de AME em crianças de 0 a 6 meses foi de 47%, enquanto o percentual de Aleitamento Materno Continuado (AMC) alcançou 53%^(10)^.

Diante da fragilidade do cenário do AME no Estado do Maranhão, esta pesquisa enfatiza a importância da educação em saúde como forma de incentivo ao AM em todos os níveis de atenção. Nesse sentido, identificar o nível de letramento em saúde de mulheres atendidas no Banco de Leite Humano (BLH) de um Hospital público universitário, no estado do Maranhão, contribui para futuras intervenções que promovam tanto o AME quanto o AMC. A observação da carência de dados sobre essa temática no Brasil, especialmente na região Nordeste^(5)^, motivou a formulação da seguinte questão da pesquisa: o Letramento em Saúde contribui para a promoção do Aleitamento Materno Exclusivo?

Embora o presente estudo, de natureza transversal, identifique associações significativas entre letramento funcional em saúde e variáveis relacionadas à amamentação, não é possível afirmar causalidade entre os fatores. Ainda assim, os achados sugerem caminhos relevantes para futuras pesquisas e intervenções em saúde pública.

O LS desempenha um papel importante na melhoria dos serviços de saúde e na obtenção de resultados positivos para a vida da cidadania moderna. Trata-se de uma combinação essencial de capital social que deve ser vista como uma política integrada, não apenas no setor de saúde, mas também em outros setores^(2)^. O LS é essencial para o avanço de políticas públicas em nível local, nacional e global, promovendo a melhoria do conhecimento sobre a amamentação entre as mulheres lactantes^(6)^.

Neste contexto, o estudo teve como objetivo descrever e analisar o letramento funcional em saúde das mulheres lactantes atendidas em banco de leite humano no estado do Maranhão.

## MÉTODO

### Tipo do Estudo

Este é um estudo transversal, descritivo.

O estudo transversal, em particular, é empregado para mensurar a prevalência de características, comportamentos e condições de saúde de uma determinada população. Como os dados são coletados apenas uma vez, esse tipo de estudo é útil para compreender a distribuição de uma situação em uma amostra e identificar associações entre variáveis. Contudo, não permite determinar relações de causa e efeito, visto que não há acompanhamento ao longo do tempo^(11)^.

O método quantitativo tem como foco o estudo de níveis mensuráveis da realidade, com o objetivo de identificar e interpretar dados, indicadores e tendências observáveis e analisáveis de forma objetiva^(12)^.

### Local do Estudo

O cenário da pesquisa foi o Banco de Leite Humano do hospital público universitário da região Nordeste do Brasil, estado do Maranhão. A instituição é certificada como hospital de ensino e reconhecida como Hospital Amigo da Criança, integrando a Rede Nacional de Bancos de Leite Humano e seguindo as diretrizes da Agência Nacional de Vigilância Sanitária (Anvisa). O BLH conta com equipe multiprofissional e estrutura adequada para assistência às mulheres lactantes, incluindo consultórios, sala de coleta, sala de palestras, espaço para processamento e pasteurização do leite humano, além de atendimento à beira-leito no Alojamento Conjunto. O serviço realiza atendimento diário, tanto por demanda espontânea quanto mediante agendamento, com média mensal de 38 nutrizes atendidas e cerca de 420 atendimentos no total.

### População e Critérios de Seleção

Participaram do presente estudo 187 mulheres lactantes atendidas na unidade hospitalar supracitada. Aceitaram participar de forma voluntária a responder os questionários do estudo. Os seguintes critérios de inclusão foram considerados para a definição dos participantes: ser nutriz com idade igual ou superior a 18 anos, ser atendida pela equipe do Banco de Leite Humano do hospital público universitário da região Nordeste do Brasil na Sala de Coleta ou diretamente no Banco de Leite Humano, estar amamentando bebês de até 6 meses de idade, e possuir capacidade cognitiva e comunicativa, conforme Mini Exame do Estado Mental (MEEM), para compreender e responder às perguntas relacionadas ao letramento em saúde e ao aleitamento materno. Durante o recrutamento das participantes, 15 mulheres lactantes foram excluídas por apresentarem resultados abaixo dos escores recomendados no MEEM, sendo que um escore normal é ≥ 25 pontos, perda cognitiva leve varia de 21 a 24 pontos, perda cognitiva moderada de 10 a 20 pontos e perda cognitiva grave é ≤ 9 pontos. Apenas as mulheres lactantes com escores normais ou com perda cognitiva leve foram incluídas no estudo, resultando em 187 mulheres lactantes elegíveis.

### Cálculo do Tamanho da Amostra

O tamanho amostral foi calculado considerando uma proporção estimada de 50% para o desfecho principal, nível de confiança de 95% (Z = 1,96) e margem de erro de 5% (0,05), o que resultou em uma amostra mínima necessária de 187 mulheres lactantes. Considerando uma média de 30 atendimentos mensais na unidade de saúde, a amostragem foi realizada de forma aleatória a partir de consultas aos prontuários das pacientes atendidas. Ao todo, foram entrevistadas 202 mulheres. No entanto, 15 participantes foram excluídas por não atenderem aos critérios de inclusão, totalizando uma amostra final de 187 mulheres, número que supera o mínimo necessário definido pelo cálculo amostral, conferindo maior robustez e confiabilidade aos resultados obtidos.

### Coleta de Dados

Os dados foram coletados entre agosto de 2023 e março do ano de 2024 no Banco de Leite Humano do hospital universitário no estado do Maranhão. A equipe responsável pela aplicação dos instrumentos foi composta pela pesquisadora principal, uma estudante de graduação e uma de mestrado, que receberam treinamento prévio para padronizar a aplicação, esclarecer dúvidas e garantir aspectos éticos, assegurando a qualidade dos dados.

A coleta ocorreu de segunda a sexta-feira, das 8h às 17h, horário de funcionamento do BLH. As mulheres lactantes eram abordadas aleatoriamente na sala de espera, onde os objetivos da pesquisa eram explicados. As participantes que concordaram assinavam o Termo de Consentimento Livre e Esclarecido (TCLE) antes da aplicação dos instrumentos.

Esse momento deu-se em três partes. Primeiro, com a coleta de dados sociodemográfico por meio de um questionário semiestruturado elaborado a partir do estudo de Suárez-Cotelo^(13)^. Nele foram contempladas as seguintes características demográficas: idade, estado civil, nível de escolaridade, renda mensal e município de residência. Segundo, foi aplicado o instrumento do Mini Exame do Estado Mental (MEEM)^(14)^ para avaliação do estado mental e cognitivo. Como instrumento de pesquisa, tem sido empregado em estudos epidemiológicos populacionais, integrando diversas baterias neuropsicológicas^(15)^. O instrumento considera a seguinte classificação cognitiva: normal, ≥ 25 pontos; perda cognitiva leve varia de 21 a 24 pontos; perda cognitiva moderada, de 10 a 20 pontos; e perda cognitiva grave, ≤ 9 pontos. Terceiro, foi aplicado o questionário Short Assessment Of Health Literacy for Portuguese-Speaking Adults, SAHLPA-18^(16)^, um instrumento que visa avaliar a alfabetização dos indivíduos em ambientes de saúde, classificando-a em inadequada (escore entre 0-14) ou adequada (escore > 15).

### Análise e Tratamento dos Dados

Os dados foram tabulados no Microsoft Excel 2021 e analisados no RStudio (versão 2023.12.1). Para verificar a associação entre as variáveis, utilizou-se o teste do Qui-quadrado, considerando o letramento em saúde e o Mini Exame do Estado Mental como variáveis dependentes, e a idade como variável independente, adotando-se nível de significância de p < 0,05. Devido à presença de categorias com frequência esperada inferior a cinco, as associações entre escolaridade, renda e letramento em saúde foram avaliadas pelo teste exato de Fisher. As proporções foram acompanhadas pelos respectivos intervalos de confiança de 95%, calculados pelo método exato de Wilson. Os resultados foram organizados em tabelas e discutidos à luz da literatura científica pertinente à temática.

### Aspectos Éticos

O estudo atendeu às questões éticas relacionadas aos seres humanos, em conformidade com a Resolução n.º 466/12 e Resolução n.º 510/16, do Conselho Nacional de Saúde. Este estudo é parte integrante do projeto guarda-chuva “Suficiência do leite materno: conhecimento de nutrizes”, aprovado pela Comissão Científica do Hospital Universitário da UFMA e pelo Comitê de Ética em Pesquisa da Universidade Federal do Maranhão, via Plataforma Brasil, sob Parecer n.º 5.310.907. Todas as participantes receberam orientações e esclarecimentos acerca da pesquisa, bem como o TCLE, que foi devidamente assinado após o aceite em participar da pesquisa.

A redação deste estudo seguiu as recomendações da diretriz STROBE para estudos observacionais.

## RESULTADOS

Foram incluídas na análise 187 mulheres lactantes. O grupo estudado foi caracterizado quanto aos condicionantes sociodemográficos e de saúde (Tabela 1 e 2). Em relação à idade, a maior parte das participantes, 45,46% (85), tinha entre 18 e 26 anos. Quanto ao estado civil, 40,64% (76) das mulheres lactantes eram solteiras. No que se refere à escolaridade, 54,54% (102) possuíam ensino médio completo, e 78,07% (146) sobreviviam com uma renda mensal entre 1 e 3 salários mínimos, ressaltando que o salário mínimo vigente na época da coleta era de R$ 1.320,00. Geograficamente, 86% (161) das mulheres lactantes residiam em São Luís.

**Tabela 1:** Descrição dos dados sociodemográficos das mulheres lactantes atendidas em banco de leite humano do Hospital público universitário, São Luís – MA, Brasil, 2024.

Variáveis	Frequência	
	n	%
Idade (anos)		
18 a 26	85	45,46
27 a 35	78	41,71
36 a 44	24	12,83
Estado civil		
Solteira	76	40,64
União consensual	55	29,41
Casada	50	26,74
Divorciada	5	2,68
Viúva	1	0,53
Escolaridade		
Fundamental incompleto	2	1,07
Fundamental completo	12	6,42
Médio incompleto	21	11,23
Médio completo	102	54,54
Superior incompleto	16	8,56
Superior completo	30	16,04
Pós-graduação	4	2,14
Renda		
Menor que 1 salário mínimo	20	10,7
De 1 a 3 salários mínimos	146	78,07
De 4 a 6 salários mínimos	18	9,63
Maior que 7 salários mínimos	3	1,6
Município de residência		
São Luís	161	86
Outros	23	14
**TOTAL**	**187**	**100**

**Nota**: O valor do salário-mínimo vigente no período da coleta (ago/2023 a mar/2024) era de R$ 1.320,00.

Fonte: Elaborada pela autora (2024).

**Tabela 2:** Descrição sobre informações acerca da amamentação ­anterior das mulheres lactantes atendidas em banco de leite ­humano do Hospital público universitário, São Luís – MA, Brasil, 2024.

Variáveis	Frequência	
	n	%
Recebeu orientações sobre amamentação?		
Nunca recebeu orientações	18	9,63
No pré-natal e após o parto	69	36,9
Somente após o parto	98	52,4
Somente no pré-natal	2	1,07
Experiência anterior com amamentação?		
Não, este é seu primeiro filho	98	52,4
Sim, já amamentou mais de um bebê	14	7,49
Sim, já amamentou outro bebê	75	40,11
Se já amamentou, qual o tempo?		
Nunca amamentou antes	98	52,41
< 6 meses	12	6,42
6 meses	3	1,6
> 6 meses e < 2 anos	48	25,67
> 2 anos	26	13,9
**TOTAL**	**187**	**100**

Fonte: Elaborada pela autora (2024).

Na Tabela 2, há uma variação significativa no recebimento de orientações sobre amamentação, sendo que 52,4% (98) das mulheres receberam alguma orientação sobre amamentação somente após o parto. Em relação à experiência com amamentação, 52,4% (98) das mulheres eram primíparas. Dentre aquelas que já haviam amamentado, a maioria, 25,67% (48), manteve a amamentação por um período superior a 6 meses, mas inferior a 2 anos.

A avaliação do nível de letramento em saúde das mulheres lactantes foi realizada utilizando o SAHLPA-18, frequentemente empregado em ambientes de atenção à saúde. Os resultados da pesquisa indicaram que 56,15% (105) das participantes apresentaram letramento inadequado, enquanto 43,85% (82) demonstraram letramento adequado.

Ao analisar a associação entre a função cognitiva, avaliada pelo Mini Exame do Estado Mental (MEEM), e o nível de letramento em saúde (LS), identificou-se uma associação estatisticamente significativa (Teste do Qui-quadrado = 9,13; p = 0,0025). Entre as mulheres com função cognitiva normal, 51,0% (75) apresentaram letramento inadequado (IC95%: 43,0–58,9%), enquanto 49,0% (153) apresentaram letramento adequado (IC95%: 41,1–57,0%). Já entre aquelas com perda cognitiva leve, 79,4% (7) apresentaram letramento inadequado (IC95%: 63,2–90,2%) e apenas 20,6% (34) letramento adequado (IC95%: 9,8–36,8%). Esses achados indicam que mulheres com desempenho cognitivo inferior apresentam maior frequência de letramento inadequado em saúde, em comparação com aquelas com função cognitiva preservada.

Ao analisar a associação entre faixa etária e letramento em saúde, identificou-se uma diferença estatisticamente significativa (Teste do Qui-quadrado = 9,93; p = 0,00697). Observou-se que o letramento inadequado foi mais prevalente entre mulheres mais jovens: na faixa etária de 18 a 26 anos, 67,1% (57) apresentaram letramento inadequado (IC95%: 56,3%–76,4%); entre 27 e 35 anos, 51,3% (40) (IC95%: 39,8%–62,7%); e entre 36 e 44 anos, a frequência foi de 33,3% (8) (IC95%: 17,3%–52,8%). Esses achados sugerem que o letramento em saúde tende a aumentar com a idade.

Entre as mulheres com ensino fundamental completo, 91,7% (11 em 12) apresentaram letramento inadequado (IC 95%: 61,5%–99,8%). Em contrapartida, entre aquelas com ensino superior completo, apenas 16,7% (5 em 30) apresentaram letramento inadequado (IC 95%: 5,6%–36,7%), indicando uma associação significativa entre escolaridade e nível de letramento em saúde (teste exato de Fisher, p < 0,001).

Entre as mulheres com renda inferior a 1 salário mínimo, 95,0% (19 em 20) apresentaram letramento inadequado (IC 95%: 75,1%–99,9%). Já entre aquelas com renda de 4 a 6 salários mínimos, 33,3% (6 em 18) apresentaram letramento inadequado (IC 95%: 13,3%–59,0%). A associação entre renda familiar e nível de letramento em saúde foi estatisticamente significativa (teste exato de Fisher, p = 0,0007).

## DISCUSSÃO

Características sociodemográficas influenciam diretamente o nível de LS entre mulheres lactantes, aspecto que se mostrou relevante para compreensão do perfil desse grupo. O estudo revelou uma predominância de mulheres jovens adultas, entre as mulheres lactantes avaliadas, das quais a maioria é solteira, possui ensino médio completo e aqui vale ressaltar que, embora o LS seja interdependente da escolaridade, mesmo nesta amostra com predominância de mulheres lactantes com ensino médio, o LS foi inadequado. A maioria apresenta uma renda familiar entre 1 e 3 salários mínimos. Esses dados reforçam a importância de adequar as ações de educação em saúde às necessidades específicas dessa população. Além disso, o LS é influenciado por estruturas sociais mais complexas do que aquelas descritas apenas pelo nível de escolaridade^(5)^.

Esses achados estão alinhados com outras pesquisas realizadas no Brasil, como o estudo em Altamira-PA, que analisou o LFS de usuários da Atenção Primária em Saúde, identificando idade, escolaridade e renda como fatores preditivos do LFS da população^(6)^. Outra pesquisa, focada na prevenção do câncer de mama, também evidenciou que variáveis como idade, renda e escolaridade influenciam significativamente a adoção de práticas preventivas e hábitos saudáveis entre mulheres^(17)^.

Esses dados indicam que os fatores sociodemográficos, como faixa etária, nível de escolaridade e contexto econômico, afetam diretamente o estilo de vida das mulheres lactantes e, consequentemente, o bem-estar do binômio mãe-bebê. Portanto, ao planejar ações de educação em saúde, é fundamental que as intervenções sejam adaptadas às especificidades de cada grupo. Essa abordagem pode melhorar o impacto das estratégias de educação em saúde, promovendo um cuidado mais eficaz e sustentável para mães e bebês^(18)^.

Em relação à experiência prévia de amamentação das mulheres lactantes, percebe-se que a maioria das mulheres recebeu orientações apenas após o parto. Esse dado revela um déficit na preparação durante o período pré-natal, evidenciando a necessidade de aprimorar as intervenções voltadas ao aleitamento materno. Garantir que as mulheres lactantes recebam orientações tanto antes quanto após o parto é fundamental para promover práticas adequadas desde o início da amamentação, possibilitando o conhecimento dos benefícios do AME e prevenindo as consequências da não oferta até o sexto mês de vida do bebê^(19)^.

Um estudo realizado em Imperatriz, Maranhão, verificou que 43,9% das mulheres receberam orientações sobre amamentação apenas na maternidade, evidenciando o impacto dos antecedentes obstétricos na autoeficácia em amamentar e a necessidade de reforçar o aconselhamento durante o pré-natal^(20)^. Esse estudo é corroborado em estudo internacional, que demonstrou que intervenções educativas tanto no pré-natal quanto no pós-parto são eficazes para aumentar a autoconfiança e a eficácia na amamentação. Tais dados reforçam a importância de um acompanhamento pré-natal abrangente e educativo, que ofereça o suporte necessário para que as mulheres lactantes iniciem a amamentação de forma segura e eficaz^(21)^.

Com base nos dados obtidos nesse estudo, a maioria das mulheres analisadas não tinha experiência prévia com amamentação, em grande parte por serem primíparas. A literatura indica que o sucesso na amamentação está frequentemente associado ao número de gestações e partos, uma vez que estudos indicam que mães multíparas tendem a ter maior autoconfiança e eficácia na amamentação^(20)^.

Com a aplicação do SAHLPA-18, destacaram-se, nesta pesquisa, mulheres lactantes que apresentavam LS inadequado. Os resultados obtidos corroboram outro estudo realizado no BLH do hospital público universitário da região Nordeste do Brasil, no qual o instrumento de avaliação foi a Escala de Letramento em Saúde (Health Literacy Scale – HLS-14), e 52% das puérperas também apresentaram LS inadequado^(22)^. Embora ambos os estudos revelem um alto índice de LS inadequado, a principal diferença reside no instrumento utilizado: o SAHLPA-18, que foca na compreensão de termos médicos, e o HLS-14, que avalia a compreensão de informações de saúde em diferentes contextos.

Pesquisa realizada no hospital Kosar de Qazvin, no Irã, identificou que o LS das mulheres lactantes era geralmente satisfatório, enquanto outro estudo, do mesmo país, realizado em centros de atenção integral à saúde na cidade de Rafsanjan, indicou que 71,8% das mães tinham LS de adequado a excelente^(23)^. Em contraste outros achados no mesmo país evidenciaram que a maioria das mulheres lactantes tinha LS baixo^(24)^.

A função cognitiva e o LS são elementos essenciais para o bem-estar de mulheres lactantes, influenciando diretamente sua capacidade de compreender e aplicar informações de saúde durante a gravidez e o período pós-parto. Os resultados desse estudo corroboram os achados de uma pesquisa que analisou o nível de LS e o desempenho cognitivo em um grupo de gestantes e puérperas. Esse estudo revelou que 41,6% das mulheres apresentavam dificuldades em compreender e lidar com informações relacionadas à saúde, especialmente aquelas com menor escolaridade, mais jovens e não brancas. Além disso, observou-se que mulheres no pós-parto apresentavam maior probabilidade de desempenho reduzido em aspectos como velocidade de raciocínio e resolução de problemas. Esses achados destacam a importância de considerar fatores sociais e biológicos ao desenvolver estratégias para avaliar o entendimento de saúde e a cognição em grupos vulneráveis^(25)^.

A associação entre o resultado do MEEM e o LS demonstra que, mesmo com um declínio cognitivo sutil, há uma tendência de diminuição do LS. Essa relação é particularmente perceptível em adultos mais velhos, nos quais a redução de funções como memória e flexibilidade mental pode prever uma menor capacidade de compreender e gerenciar informações relacionadas à saúde^(26)^.

O nível de LS apresentou menor desempenho entre mulheres lactantes na faixa etária de 18 a 26 anos. Essa associação foi reforçada pelo estudo realizado no Hospital Sivas Numune, na Turquia, que evidenciou a limitação do LS, especialmente entre “mães de primeira viagem”^(27)^. Apesar de terem acesso a um grande volume de informações sobre saúde, muitas dessas mães relataram sentir-se sobrecarregadas e assustadas com o excesso de informações, o que pode dificultar a tomada de decisões informadas. A idade, portanto, é um fator relevante que influencia o LS das mulheres lactantes, sendo que mães primíparas e mais jovens enfrentam desafios significativos para compreender e aplicar informações de saúde de maneira eficaz.

Observou-se uma relação significativa entre a idade materna e o LS, com pontuações maiores para mães com idade entre 36 e 44 anos, o que está em concordância com um estudo, que também identificou escores mais elevados de LS em mulheres lactantes mais velhas. Esse efeito pode estar relacionado à maior experiência das mães nessa faixa etária. De modo geral, o aumento da idade materna está associado a escores mais elevados de LS, o que, por sua vez, tende a favorecer uma maior duração e probabilidade de amamentação^(2,28,29)^.

Com base nos dados obtidos neste estudo, observa-se que o nível de escolaridade desempenha um papel crucial no LS das mulheres lactantes. A associação entre um LS adequado e níveis mais elevados de escolaridade ressalta a importância de uma trajetória educacional regular e bem-sucedida, conforme também observado pelo autor acima citado^(30)^.

Mulheres com maior nível de escolaridade demonstram maior empenho e capacidade para amamentar de forma assertiva e prolongada. Esse comportamento pode ser atribuído ao fato de essas mulheres terem maior acesso a informações sobre os benefícios que o AME oferece tanto para a mãe quanto para o filho. Além disso, espera-se que esse fator também contribua para uma melhor execução dos cuidados com as mamas durante a gestação e o puerpério. Por outro lado, observa-se uma tendência de que mulheres lactantes com menor escolaridade enfrentem mais dificuldades nos momentos iniciais do processo de amamentação, em decorrência do menor acesso a informações e práticas preventivas^(31)^.

Em contrapartida, um estudo realizado com mulheres atendidas pela Estratégia Saúde da Família na região Sudeste do Brasil revelou que o indicador “anos completos de estudo” pode não refletir o grau de alfabetização. Isso indica que o nível educacional, por si só, não garante um LS adequado, já que, mesmo usuários com alta escolaridade podem apresentar dificuldades em compreender terminologias e procedimentos específicos do contexto da saúde^(5)^.

A literatura internacional indica que níveis mais baixos de LS são mais prevalentes em grupos de baixa renda. Estudo realizado no Havaí, EUA, identificou que mães vulneráveis, com LS inadequado e baixa renda, tendem a tomar decisões de saúde menos favoráveis, o que pode impactar negativamente o cuidado com a própria saúde e o bem-estar dos filhos^(32)^.

Outro estudo, realizado em Laos, país do Sudeste Asiático, identificou que o LS é inadequado tanto nas áreas urbanas quanto nas rurais do país. Além disso, destacou a renda familiar como o principal fator de influência nesse desfecho de saúde^(33)^.

Mulheres de baixa renda apresentam maior suscetibilidade a baixos níveis de LS, reforçando a associação entre LS e renda familiar. A renda, como indicador de status social, está diretamente ligada ao acesso à informação e a serviços de saúde, influenciando negativamente os níveis de LS em populações economicamente desfavorecidas^(5)^.

As limitações do estudo incluem sua natureza transversal, o uso de amostra não probabilística, o foco em um único município do Nordeste brasileiro e a ausência de uma análise qualitativa que contemple aspectos culturais das práticas de amamentação. Soma-se a isso a ausência de uma abordagem multicêntrica em nível regional ou estadual e a não inclusão de outros bancos de leite humano da região. Além disso, o grupo de mulheres lactantes participantes não representa a totalidade do público feminino, e identificou-se uma escassez de literatura científica sobre o letramento em saúde nesse público específico no contexto brasileiro. Esses fatores reforçam a necessidade de novas pesquisas que ampliem o entendimento sobre o letramento em saúde de mulheres lactantes em diferentes bancos de leite humano nas diversas regiões do país.

## CONCLUSÃO

Conclui-se que a maioria das mulheres lactantes atendidas no Banco de Leite humano no estado do Maranhão apresentam letramento funcional em saúde inadequado. Os dados demonstram uma associação significativa entre o nível de LS e fatores como idade, escolaridade, renda familiar e resultado do MEEM. Mulheres lactantes mais jovens, com menor escolaridade, renda mais baixa e sinais de perda cognitiva leve apresentaram maior prevalência de LS inadequado.

Diante dos achados deste estudo, salienta-se que o letramento em saúde, enquanto base conceitual para a promoção da saúde, configura-se como uma estratégia efetiva de cuidado à saúde das mulheres lactantes. Sua adoção como diretriz em políticas públicas pode favorecer o empoderamento, o autocuidado, e a ampliação do acesso qualificado aos serviços de saúde, contribuindo para melhoria dos desfechos relacionados à amamentação e à saúde materno-infantil.

Embora a prevalência de LS inadequado neste estudo seja elevada, sugere-se a introdução de recursos que aprimorem a comunicação entre profissionais de saúde e pacientes, especialmente no caso de mulheres lactantes com baixa renda e escolaridade. Medidas educativas são fundamentais para que os profissionais adaptem sua linguagem, ajustem orientações e prescrições e desenvolvam instrumentos de comunicação que atendam às necessidades e habilidades das mulheres lactantes com maior risco de déficits no LS.

Sugere-se a inserção do letramento em saúde na formação e capacitação permanente de profissionais da atenção primária, visando qualificar o cuidado às mulheres lactantes. Na prática assistencial, é essencial adotar estratégias de comunicação adequadas ao perfil sociocultural das usuárias. Futuras pesquisas, preferencialmente multicêntricas e qualitativas, poderão ampliar a compreensão do tema em diferentes contextos regionais.

## DISPONIBILIDADE DE DADOS

Todo o conjunto de dados que dá suporte aos resultados deste estudo foi publicado no próprio artigo.

# Translation: Alfabetización en salud entre mujeres lactantes y promoción de la lactancia materna y la salud materno-infantil

**DOI:** 10.1590/1980-220X-REEUSP-2025-0180es

**Authors:** Élida Cristina Santos Corrêa, Poliana Pereira Costa Rabelo, Pablo Nascimento Cruz, Isaura Letícia Tavares Palmeira Rolim, Katarinne Lima Moraes, Janaína Miranda Bezerra

**Affiliations:** 1 Universidade Federal do Maranhão, Programa de Pós-Graduação em Enfermagem, São Luís, MA, Brasil.; 2 Universidade Brasília Ceilândia, Faculdade de Ciências e Tecnologias em Saúde, Brasília, DF, Brasil.; 3 Universidade Federal do Maranhão, Campus Imperatriz, MA, Brasil.

**Keywords:** Alfabetización en salud, Lactancia materna, Enfermería

## Abstract

**Objetivo:**

Describir y analizar la alfabetización funcional en salud de mujeres lactantes atendidas en un Banco de Leche Humana de Maranhão.

**Método:**

Estudio transversal y descriptivo, realizado entre agosto de 2023 y marzo de 2024, en el Banco de Leche de un hospital universitario público del noreste de Brasil, utilizando una escala validada de alfabetización en salud.

**Resultados:**

Participaron 187 mujeres lactando, de las cuales el 56,15 % presentaba una alfabetización en salud inadecuada. Hubo una asociación significativa entre la alfabetización en salud y el Mini Examen del Estado Mental, con mayor probabilidad de alfabetización inadecuada entre las mujeres más jóvenes (67.1 %), con educación primaria completa (91.7 %), secundaria incompleta (91.7 %) e ingresos inferiores al salario mínimo (95 %). Se aplicaron pruebas de asociación (Chi-cuadrado) con significación en p < 0,05, destacando la asociación entre alfabetización y edad (p = 0,00697).

**Conclusión:**

La mayoría de las lactantes evaluadas presentaron una alfabetización en salud inadecuada, lo que evidencia la importancia de las estrategias educativas como base para la promoción de la salud y la cualificación de la atención prestada a este público.

## INTRODUCCIÓN

La alfabetización en salud (AS) está asociada con la comprensión personal, el incentivo y la capacidad de tomar decisiones relacionadas con la salud a lo largo de la vida. Algunas de estas capacidades incluyen el conocimiento de los números, la lectura, la escritura y la competencia para expresar puntos de vista, comunicarse de manera eficaz y utilizar tecnologías. En este contexto, la AS se ha vuelto esencial para la promoción de la salud y la mejora en la toma de decisiones en materia de salud^(1)^.

Por otro lado, cuando este nivel es bajo, los impactos pueden ser negativos, como en el caso de la lactancia materna (LM). Las madres con AS bajo tienden a tener menos comprensión y acceso a la información sobre la lactancia materna, lo que puede perjudicar la continuidad de la LM. Por lo tanto, el conocimiento sobre los beneficios de la lactancia materna, tanto para la madre como para el bebé, es esencial para el éxito de esta práctica^(2)^.

Estudios internacionales, como el de Valero-Chillerón et al.^(3)^, realizado en Valencia, España, y el de Fatmawati et al.^(4)^, en Malang, Indonesia, analizaron la influencia de la alfabetización en salud en la lactancia materna exclusiva hasta los seis meses de vida. Además, investigaciones desarrolladas en hospitales españoles se han centrado en la creación de instrumentos específicos para evaluar la alfabetización orientada a la lactancia materna. Estos estudios refuerzan la necesidad de realizar más investigaciones en México sobre el impacto de la alfabetización en salud en la práctica de la lactancia materna, teniendo en cuenta el contexto cultural y social del país^(3,4)^.

El instrumento SAHLPA-18 ha sido validado y aplicado en distintos contextos, como en el estudio de Campos et al.^(5)^, que investigó la asociación de la alfabetización funcional en salud (AFS) entre mujeres atendidas por la Estrategia de Salud Familiar, en el estado de São Paulo, y en el estudio de Lima et al.^(6)^, que analizaron el nivel de alfabetización funcional en usuarios de la atención primaria en el estado de Pará.

Datos más recientes muestran que la tasa global de lactancia materna exclusiva (LME) hasta los 6 meses alcanzó el 48 %, acercándose al objetivo del 50 % para el año 2025. Sin embargo, existen disparidades regionales: Ruanda alcanzó el 87 %, Sri Lanka el 82 %, Pakistán el 37 % y Estados Unidos el 25 %. Factores como las políticas de apoyo a la lactancia materna y la educación influyen en estas tasas, así como los permisos de maternidad cortos^(7,8)^.

En Brasil, las tasas de LME han aumentado a lo largo de los años, destacando los esfuerzos en políticas públicas orientadas a la promoción, protección y apoyo a la lactancia materna. Programas como la Iniciativa Hospital Amigo de la Niñez (IHAC en la sigla en portugués) y la Estrategia Amamanta y Alimenta Brasil, junto con la ampliación del acceso a la información y la creación de bancos de leche materna, han contribuido a este avance. Según el Estudio Nacional de Alimentación y Nutrición Infantil (ENANI), en 2020 se constató una tasa de LME del 45,7 % en niños menores de seis meses^(9)^.

En el estado de Maranhão, según el Sistema Nacional de Vigilancia Alimentaria y Nutricional (SISVAN), los datos de 2021 muestran que la tasa de LME en niños de 0 a 6 meses fue del 47 %, mientras que el porcentaje de lactancia materna continuada (LMC) alcanzó el 53 %^(10)^.

Ante la fragilidad del panorama de la LME en el estado de Maranhão, esta investigación enfatiza la importancia de la educación en salud como forma de incentivar la lactancia materna en todos los niveles de atención. En este sentido, identificar el nivel de alfabetización en salud de las mujeres atendidas en el Banco de Leche Humana (BLH) de un hospital público universitario, en el estado de Maranhão, contribuye a futuras intervenciones que promuevan tanto la LME como la LMC. La observación de la falta de datos sobre este tema en Brasil, especialmente en la región Nordeste^(5)^, motivó la formulación de la siguiente pregunta de investigación: ¿Contribuye la alfabetización en salud a la promoción de la lactancia materna exclusiva?

Aunque el presente estudio, de naturaleza transversal, identifica asociaciones significativas entre la alfabetización funcional en salud y variables relacionadas con la lactancia materna, no es posible afirmar la causalidad entre los factores. Aun así, los hallazgos sugieren caminos relevantes para futuras investigaciones e intervenciones en salud pública.

La AS desempeña un papel importante en la mejora de los servicios de salud y en la obtención de resultados positivos para la vida de la ciudadanía moderna. Se trata de una combinación esencial de capital social que debe considerarse como una política integrada, no solo en el sector de la salud, sino también en otros sectores^(2)^. La AS es esencial para el avance de las políticas públicas a nivel local, nacional y mundial, promoviendo la mejora del conocimiento sobre la lactancia materna entre las mujeres lactantes^(6)^.

En este contexto, el estudio tuvo como objetivo describir y analizar la alfabetización funcional en salud de las mujeres lactantes atendidas en un banco de leche humana en el estado de Maranhão.

## MÉTODO

### Tipo de Estudio

Este es un estudio transversal y descriptivo.

El estudio transversal, en particular, se emplea para medir la prevalencia de características, comportamientos y condiciones de salud de una población determinada. Como los datos se recogen una sola vez, este tipo de estudio es útil para comprender la distribución de una situación en una muestra e identificar asociaciones entre variables. Sin embargo, no permite determinar relaciones de causa y efecto, ya que no hay un seguimiento a lo largo del tiempo^(11)^.

El método cuantitativo se centra en el estudio de niveles medibles de la realidad, con el objetivo de identificar e interpretar datos, indicadores y tendencias observables y analizables de forma objetiva^(12)^.

### Lugar del Estudio

El escenario de la investigación fue el Banco de Leche Humana del hospital público universitario de la región noreste de Brasil, en el estado de Maranhão. La institución está certificada como hospital universitario y reconocida como Hospital Amigo de la Niñez, integra la Red Nacional de Bancos de Leche Humana y sigue las directrices de la Agencia Nacional de Vigilancia Sanitaria (Anvisa). El BLH cuenta con un equipo multidisciplinario y una estructura adecuada para atender a las mujeres lactantes, incluyendo consultorios, sala de recolección, sala de conferencias, espacio para el procesamiento y pasteurización de la leche materna, además de atención a pie de cama en el Alojamiento Conjunto. El servicio atiende diariamente, tanto por demanda espontánea como con cita previa, con un promedio mensual de 38 madres lactantes atendidas y alrededor de 420 atendidos en total.

### Población y Criterios de Selección

Participaron en el presente estudio 187 mujeres lactantes atendidas en la unidad hospitalaria mencionada. Aceptaron participar de forma voluntaria en la respuesta a los cuestionarios del estudio. Se consideraron los siguientes criterios de inclusión para definir a las participantes: ser lactante mayor de 18 años, ser atendida por el equipo del Banco de Leche Humana del hospital público universitario de la región noreste de Brasil en la Sala de Recolección o directamente en el Banco de Leche Humana, estar amamantando bebés de hasta 6 meses de edad y poseer capacidad cognitiva y comunicativa, de acuerdo con el Mini Examen del Estado Mental (MEEM), para comprender y responder a las preguntas relacionadas con la alfabetización en salud y la lactancia materna. Durante el reclutamiento de las participantes, se excluyó a 15 mujeres lactantes por presentar resultados por debajo de las puntuaciones recomendadas en el MEEM, siendo una puntuación normal ≥ 25 puntos, una pérdida cognitiva leve de 21 a 24 puntos, una pérdida cognitiva moderada de 10 a 20 puntos y una pérdida cognitiva grave ≤ 9 puntos. Solo se incluyeron en el estudio las mujeres lactantes con puntuaciones normales o con pérdida cognitiva leve, lo que dio como resultado 187 mujeres lactantes elegibles.

### Cálculo del Tamaño de la Muestra

El tamaño de la muestra se calculó considerando una proporción estimada del 50 % para el resultado principal, un nivel de confianza del 95 % (Z = 1,96) y un margen de error del 5 % (0,05), lo que dio como resultado una muestra mínima necesaria de 187 mujeres lactantes. Teniendo en cuenta una media de 30 consultas mensuales en el centro de salud, el muestreo se realizó de forma aleatoria a partir de la consulta de los expedientes de las pacientes atendidas. En total, se entrevistó a 202 mujeres. Sin embargo, se excluyó a 15 participantes por no cumplir los criterios de inclusión, lo que dio lugar a una muestra final de 187 mujeres, cifra que supera el mínimo necesario definido por el cálculo muestral, lo que confiere mayor solidez y fiabilidad a los resultados obtenidos.

### Recolección de Datos

Los datos se recopilaron entre agosto de 2023 y marzo de 2024 en el Banco de Leche Humana del hospital universitario del estado de Maranhão. El equipo responsable de la aplicación de los instrumentos estuvo compuesto por la investigadora principal, una estudiante de licenciatura y otra de maestría, quienes recibieron capacitación previa para estandarizar la aplicación, aclarar dudas y garantizar los aspectos éticos, asegurando la calidad de los datos.

La recolección se realizó de lunes a viernes, de 8 a. m. a 5 p. m., horario de atención del BLH. Las mujeres lactantes fueron abordadas al azar en la sala de espera, donde se les explicaron los objetivos de la investigación. Las participantes que aceptaron firmaron el Término de Consentimiento Libre y Esclarecido (TCLE) antes de la aplicación de los instrumentos.

Este momento se dividió en tres partes. En primer lugar, se recogieron datos sociodemográficos mediante un cuestionario semiestructurado elaborado a partir del estudio de Suárez-Cotelo^(13)^. En él se contemplaron las siguientes características demográficas: edad, estado civil, nivel de estudios, ingresos mensuales y municipio de residencia. En segundo lugar, se aplicó el instrumento Mini Examen del Estado Mental (MEEM)^(14)^ para evaluar el estado mental y cognitivo. Como instrumento de investigación, se ha utilizado en estudios epidemiológicos poblacionales, integrando diversas baterías neuropsicológicas^(15)^. El instrumento considera la siguiente clasificación cognitiva: normal, ≥ 25 puntos; pérdida cognitiva leve, de 21 a 24 puntos; pérdida cognitiva moderada, de 10 a 20 puntos; y pérdida cognitiva grave, ≤ 9 puntos. En tercer lugar, se aplicó el cuestionario Short Assessment Of Health Literacy for Portuguese-Speaking Adults, SAHLPA-18^(16)^, un instrumento que tiene como objetivo evaluar la alfabetización de los individuos en entornos de salud, clasificándola como inadecuada (puntuación entre 0 y 14) o adecuada (puntuación > 15).

### Análisis y Tratamiento de los Datos

Los datos se tabularon en Microsoft Excel 2021 y se analizaron en RStudio (versión 2023.12.1). Para verificar la asociación entre las variables, se utilizó la prueba de Chi-cuadrado, considerando la alfabetización en salud y el Mini Examen del Estado Mental como variables dependientes, y la edad como variable independiente, adoptando un nivel de significación de p < 0,05. Debido a la presencia de categorías con una frecuencia esperada inferior a cinco, las asociaciones entre escolaridad, ingresos y alfabetización en salud se evaluaron mediante la prueba exacta de Fisher. Las proporciones se acompañaron de los respectivos intervalos de confianza del 95 %, calculados mediante el método exacto de Wilson. Los resultados se organizaron en tablas y se discutieron a la luz de la literatura científica pertinente al tema.

### Aspectos Éticos

El estudio cumplió con las cuestiones éticas relacionadas con los seres humanos, de conformidad con la Resolución n.º 466/12 y la Resolución n.º 510/16 del Consejo Nacional de Salud. Este estudio forma parte del proyecto marco «Suficiencia de la leche materna: conocimiento de las madres lactantes», aprobado por la Comisión Científica del Hospital Universitario de la UFMA y por el Comité de Ética en Investigación de la Universidad Federal de Maranhão, a través de la Plataforma Brasil, con el dictamen n.º 5.310.907. Todas las participantes recibieron orientación y aclaraciones sobre la investigación, así como el TCLE, que fue debidamente firmado tras aceptar participar en la investigación.

La redacción de este estudio siguió las recomendaciones de la directriz STROBE para estudios observacionales.

## RESULTADOS

Se incluyeron en el análisis 187 mujeres lactantes. El grupo estudiado se caracterizó en función de los factores sociodemográficos y de salud (Tablas 1 y 2). En cuanto a la edad, la mayor parte de las participantes, el 45,46 % (85), tenían entre 18 y 26 años. En cuanto al estado civil, el 40,64 % (76) de las mujeres lactantes eran solteras. En cuanto a la escolaridad, el 54,54 % (102) tenían la educación secundaria completa, y el 78,07 % (146) sobrevivían con un ingreso mensual entre 1 y 3 salarios mínimos, destacando que el salario mínimo vigente en el momento de la recolección era de R$ 1.320,00. Geográficamente, el 86 % (161) de las mujeres lactantes residían en São Luis.

**Tabla 1:** Descripción de los datos sociodemográficos de las mujeres lactantes atendidas en el banco de leche humana de un hospital universitario público, São Luís – MA, Brasil, 2024.

Variables	Frecuencia	
	n	%
Edad (años)		
18 a 26	85	45,46
27 a 35	78	41,71
36 a 44	24	12,83
Estado civil		
Soltero	76	40,64
Unión consensual	55	29,41
Casado	50	26,74
Divorciado	5	2,68
Viudo	1	0,53
Escolarización		
Estudios primarios incompletos	2	1,07
Enseñanza primaria completa	12	6,42
Secundario incompleto	21	11,23
Secundario completo	102	54,54
Título universitario incompleto	16	8,56
Titulación universitaria completa	30	16,04
Postgrado	4	2,14
Ingresos		
Menos de 1 salario mínimo	20	10,7
De 1 a 3 salarios mínimos	146	78,07
De 4 a 6 salarios mínimos	18	9,63
Más de 7 salarios mínimos	3	1,6
Municipio de residencia		
São Luís	161	86
Otros	23	14
**TOTAL**	**187**	**100**

**Nota**: El valor del salario mínimo vigente durante el período de recolección (Ago/2023 a Mar/2024) fue de R$1.320,00.

Fuente: Elaboración propia (2024).

**Tabla 2:** Descripción de la información sobre lactancia materna previa entre las mujeres lactantes atendidas en el banco de leche humana de un hospital universitario público, São Luís – MA, Brasil, 2024.

Variables	Frecuencia	
	n	%
¿Recibió orientación sobre lactancia materna?		
Nunca recibió orientación	18	9,63
Durante el prenatal y después del parto	69	36,9
Sólo después del parto	98	52,4
Sólo durante el prenatal	2	1,07
¿Experiencia previa con la lactancia materna?		
No, es su primer hijo	98	52,4
Sí, ha amamantado a más de un bebé	14	7,49
Sí, ha amamantado a otro bebé	75	40,11
Si ha amamantado, ¿durante cuánto tiempo?		
Nunca había dado el pecho	98	52,41
< 6 meses	12	6,42
6 meses	3	1,6
> 6 meses y < 2 años	48	25,67
> 2 años	26	13,9
**TOTAL**	**187**	**100**

Fuente: Elaboración propia (2024).

La Tabla 2 muestra una variación significativa en la recepción de orientaciones sobre amamantamiento, con 52,4% (98) de las mujeres recibiendo alguna orientación sobre amamantamiento solamente después del parto. Respecto a la experiencia en lactancia, el 52,4% (98) de las mujeres eran primíparas. De las que ya habían amamantado, la mayoría, 25,67% (48), habían mantenido la lactancia durante más de 6 meses, pero menos de 2 años.

La evaluación del nivel de alfabetización en salud de las mujeres lactantes se realizó utilizando el SAHLPA-18, frecuentemente empleado en entornos de atención sanitaria. Los resultados de la investigación indicaron que el 56,15 % (105) de las participantes presentaban una alfabetización inadecuada, mientras que el 43,85 % (82) mostraban una alfabetización adecuada.

Al analizar la asociación entre la función cognitiva, evaluada mediante el Mini Examen del Estado Mental (MEEM), y el nivel de alfabetización en salud (AS), se identificó una asociación estadísticamente significativa (prueba de Chi-cuadrado = 9,13; p = 0,0025). Entre las mujeres con función cognitiva normal, el 51,0 % (75) presentaban un nivel de alfabetización inadecuado (IC 95 %: 43,0-58,9 %), mientras que el 49,0 % (153) presentaban un nivel adecuado (IC 95 %: 41,1-57,0 %). Entre aquellas con pérdida cognitiva leve, el 79,4 % (7) presentaban un nivel de alfabetización inadecuado (IC del 95 %: 63,2-90,2 %) y solo el 20,6 % (34) un nivel adecuado (IC del 95 %: 9,8-36,8 %). Estos hallazgos indican que las mujeres con un rendimiento cognitivo inferior presentan una mayor frecuencia de alfabetización inadecuada en salud, en comparación con aquellas con función cognitiva preservada.

Al analizar la asociación entre el grupo de edad y la alfabetización en salud, se identificó una diferencia estadísticamente significativa (prueba de Chi-cuadrado = 9,93; p = 0,00697). Se observó que la alfabetización inadecuada era más prevalente entre las mujeres más jóvenes: en el grupo de edad de 18 a 26 años, el 67,1 % (57) presentaba alfabetización inadecuada (IC del 95 %: 56,3 %-76,4 %); entre 27 y 35 años, el 51,3 % (40) (IC95%: 39,8 %–62,7 %); y entre 36 y 44 años, la frecuencia fue del 33,3 % (8) (IC95%: 17,3 %–52,8 %). Estos hallazgos sugieren que la alfabetización en salud tiende a aumentar con la edad.

Entre las mujeres con educación primaria completa, el 91,7 % (11 de 12) presentaban una alfabetización inadecuada (IC 95 %: 61,5 %-99,8 %). Por el contrario, entre las que tenían estudios superiores completos, solo el 16,7 % (5 de 30) presentaban un nivel de alfabetización inadecuado (IC 95 %: 5,6 %-36,7 %), lo que indica una asociación significativa entre el nivel educativo y el nivel de alfabetización en salud (prueba exacta de Fisher, p < 0,001).

Entre las mujeres con ingresos inferiores a 1 salario mínimo, el 95,0 % (19 de 20) presentaban un nivel de alfabetización inadecuado (IC del 95 %: 75,1 %-99,9 %). Entre aquellas con ingresos de 4 a 6 salarios mínimos, el 33,3 % (6 de 18) presentaban un nivel de alfabetización inadecuado (IC 95 %: 13,3 %-59,0 %). La asociación entre los ingresos familiares y el nivel de alfabetización en salud fue estadísticamente significativa (prueba exacta de Fisher, p = 0,0007).

## DISCUSIÓN

Las características sociodemográficas influyen directamente en el nivel de AS entre las mujeres lactantes, aspecto que resultó relevante para comprender el perfil de este grupo.

El estudio reveló un predominio de mujeres adultas jóvenes entre las mujeres lactantes evaluadas, la mayoría de las cuales son solteras y tienen estudios secundarios completos, y cabe destacar que, aunque la AS es interdependiente del nivel educativo, incluso en esta muestra con predominio de mujeres lactantes con estudios secundarios, la AS era inadecuada. La mayoría presenta un ingreso familiar entre 1 y 3 salarios mínimos. Estos datos refuerzan la importancia de adaptar las acciones de educación en salud a las necesidades específicas de esta población. Además, la AS está influenciada por estructuras sociales más complejas que las descritas únicamente por el nivel de escolaridad^(5)^.

Estos hallazgos están en consonancia con otras investigaciones realizadas en Brasil, como el estudio en Altamira-PA, que analizó el LFS de los usuarios de la Atención Primaria en Salud, identificando la edad, la escolaridad y los ingresos como factores predictivos del LFS de la población ^(6)^. Otra investigación, centrada en la prevención del cáncer de mama, también evidenció que variables como la edad, los ingresos y el nivel educativo influyen significativamente en la adopción de prácticas preventivas y hábitos saludables entre las mujeres^(17)^.

Estos datos indican que los factores sociodemográficos, como el grupo de edad, el nivel educativo y el contexto económico, afectan directamente al estilo de vida de las mujeres lactantes y, en consecuencia, al bienestar del binomio madre-bebé. Por lo tanto, al planificar acciones de educación en salud, es fundamental que las intervenciones se adapten a las especificidades de cada grupo. Este enfoque puede mejorar el impacto de las estrategias de educación en salud, promoviendo una atención más eficaz y sostenible para las madres y los bebés^(18)^.

En cuanto a la experiencia previa de lactancia de las mujeres lactantes, se observa que la mayoría de ellas recibieron orientación solo después del parto. Este dato revela un déficit en la preparación durante el período prenatal, lo que evidencia la necesidad de mejorar las intervenciones dirigidas a la lactancia materna. Garantizar que las mujeres lactantes reciban orientación tanto antes como después del parto es fundamental para promover prácticas adecuadas desde el inicio de la lactancia, permitiendo conocer los beneficios de la LME y previniendo las consecuencias de la falta de lactancia hasta los seis meses de vida del bebé^(19)^.

Un estudio realizado en Imperatriz, Maranhão, verificó que el 43,9 % de las mujeres recibieron orientación sobre lactancia materna solo en la maternidad, lo que evidencia el impacto de los antecedentes obstétricos en la autoeficacia en la lactancia materna y la necesidad de reforzar la ASesoramiento durante el periodo prenatal^(20)^. Este estudio se ve corroborado por un estudio internacional, que demostró que las intervenciones educativas tanto en el periodo prenatal como en el posparto son eficaces para aumentar la autoconfianza y la eficacia en la lactancia materna. Estos datos refuerzan la importancia de un seguimiento prenatal integral y educativo, que ofrezca el apoyo necesario para que las mujeres lactantes inicien la lactancia materna de forma segura y eficaz^(21)^.

Según los datos obtenidos en este estudio, la mayoría de las mujeres analizadas no tenían experiencia previa en lactancia materna, en gran parte por ser primíparas. La literatura indica que el éxito en la lactancia materna se asocia frecuentemente con el número de gestaciones y partos, ya que los estudios indican que las madres multíparas tienden a tener mayor autoconfianza y eficacia en la lactancia materna^(20)^.

Con la aplicación del SAHLPA-18, en esta investigación se destacaron las mujeres lactantes que presentaban AS inadecuada. Los resultados obtenidos corroboran otro estudio realizado en el BLH del hospital público universitario de la región Nordeste de Brasil, en el que el instrumento de evaluación fue la Escala de Alfabetización en Salud (Health Literacy Scale – HLS-14), y el 52 % de las puérperas también presentaron AS inadecuada^(22)^. Aunque ambos estudios revelan un alto índice de AS inadecuado, la principal diferencia radica en el instrumento utilizado: el SAHLPA-18, que se centra en la comprensión de términos médicos, y el HLS-14, que evalúa la comprensión de la información sanitaria en diferentes contextos.

Una investigación realizada en el hospital Kosar de Qazvin, en Irán, identificó que la AS de las mujeres lactantes era en general satisfactoria, mientras que otro estudio, del mismo país, realizado en centros de atención integral de salud en la ciudad de Rafsanjan, indicó que el 71,8 % de las madres tenían una AS de adecuada a excelente^(23)^. En contraste, otros hallazgos en el mismo país evidenciaron que la mayoría de las mujeres lactantes tenían una AS baja^(24)^.

La función cognitiva y la AS son elementos esenciales para el bienestar de las mujeres lactantes, ya que influyen directamente en su capacidad para comprender y aplicar la información de salud durante el embarazo y el posparto. Los resultados de este estudio corroboran los hallazgos de una investigación que analizó el nivel de AS y el rendimiento cognitivo en un grupo de mujeres embarazadas y puérperas. Este estudio reveló que el 41,6 % de las mujeres tenían dificultades para comprender y manejar la información relacionada con la salud, especialmente aquellas con menor nivel educativo, más jóvenes y no blancas. Además, se observó que las mujeres en el posparto tenían más probabilidades de presentar un rendimiento reducido en aspectos como la velocidad de razonamiento y la resolución de problemas. Estos hallazgos destacan la importancia de considerar los factores sociales y biológicos al desarrollar estrategias para evaluar la comprensión de la salud y la cognición en grupos vulnerables^(25)^.

La asociación entre el resultado del MEEM y la AS demuestra que, incluso con un deterioro cognitivo sutil, existe una tendencia a la disminución dla AS. Esta relación es particularmente perceptible en adultos mayores, en los que la reducción de funciones como la memoria y la flexibilidad mental puede predecir una menor capacidad para comprender y gestionar información relacionada con la salud^(26)^.

El nivel de AS presentó un menor rendimiento entre las mujeres lactantes de entre 18 y 26 años. Esta asociación se vio reforzada por el estudio realizado en el Hospital Sivas Numune, en Turquía, que evidenció la limitación de la AS, especialmente entre las «madres primerizas»^(27)^. A pesar de tener acceso a un gran volumen de información sobre salud, muchas de estas madres informaron sentirse abrumadas y asustadas por el exceso de información, lo que puede dificultar la toma de decisiones informadas. La edad, por lo tanto, es un factor relevante que influye en la AS de las mujeres lactantes, y las madres primíparas y más jóvenes enfrentan desafíos significativos para comprender y aplicar la información de salud de manera eficaz.

Se observó una relación significativa entre la edad materna y la AS, con puntuaciones más altas para las madres de entre 36 y 44 años, lo que concuerda con un estudio que también identificó puntuaciones más altas de AS en mujeres lactantes de más edad. Este efecto puede estar relacionado con la mayor experiencia de las madres de este grupo de edad. En general, el aumento de la edad materna se asocia con puntuaciones más altas de AS, lo que, a su vez, tiende a favorecer una mayor duración y probabilidad de lactancia materna^(2,28,29)^.

A partir de los datos obtenidos en este estudio, se observa que el nivel educativo desempeña un papel crucial en la AS de las mujeres lactantes. La asociación entre un AS adecuado y niveles más altos de escolaridad resalta la importancia de una trayectoria educativa regular y exitosa, como también lo observó el autor citado anteriormente^(30)^.

Las mujeres con un mayor nivel educativo muestran un mayor compromiso y capacidad para amamantar de forma asertiva y prolongada. Este comportamiento puede atribuirse al hecho de que estas mujeres tienen un mayor acceso a la información sobre los beneficios que la AME ofrece tanto a la madre como al hijo. Además, se espera que este factor también contribuya a una mejor ejecución de los cuidados mamarios durante el embarazo y el puerperio. Por otro lado, se observa una tendencia a que las mujeres lactantes con menor nivel educativo enfrenten más dificultades en los momentos iniciales del proceso de lactancia, debido al menor acceso a la información y a las prácticas preventivas^(31)^.

Por el contrario, un estudio realizado con mujeres atendidas por la Estrategia de Salud Familiar en la región sudeste de Brasil reveló que el indicador «años completos de estudio» puede no reflejar el grado de alfabetización. Esto indica que el nivel educativo, por sí solo, no garantiza un AS adecuado, ya que incluso los usuarios con un alto nivel de escolaridad pueden tener dificultades para comprender terminología y procedimientos específicos del contexto de la salud^(5)^.

La literatura internacional indica que los niveles más bajos de AS son más prevalentes en grupos de bajos ingresos. Un estudio realizado en Hawái, EE. UU., identificó que las madres vulnerables, con AS inadecuado y bajos ingresos, tienden a tomar decisiones de salud menos favorables, lo que puede impactar negativamente en el cuidado de su propia salud y el bienestar de sus hijos^(32)^.

Otro estudio, realizado en Laos, país del sudeste asiático, identificó que la AS es inadecuado tanto en las zonas urbanas como en las rurales del país. Además, destacó los ingresos familiares como el principal factor que influye en este resultado de salud^(33)^.

Las mujeres con bajos ingresos presentan una mayor susceptibilidad a los bajos niveles de AS, lo que refuerza la asociación entre la AS y los ingresos familiares. Los ingresos, como indicador del estatus social, están directamente relacionados con el acceso a la información y a los servicios de salud, lo que influye negativamente en los niveles de AS en las poblaciones económicamente desfavorecidas^(5)^.

Las limitaciones del estudio incluyen su naturaleza transversal, el uso de una muestra no probabilística, el enfoque en un solo municipio del noreste de México y la ausencia de un análisis cualitativo que contemple los aspectos culturales de las prácticas de lactancia materna. A esto se suma la ausencia de un enfoque multicéntrico a nivel regional o estatal y la no inclusión de otros bancos de leche materna de la región. Además, el grupo de mujeres lactantes participantes no representa la totalidad de la población femenina, y se identificó una escasez de literatura científica sobre la alfabetización en salud en este público específico en el contexto brasileño. Estos factores refuerzan la necesidad de nuevas investigaciones que amplíen la comprensión sobre la alfabetización en salud de las mujeres lactantes en diferentes bancos de leche humana en diversas regiones del país.

## CONCLUSIÓN

Se concluye que la mayoría de las mujeres lactantes atendidas en el Banco de Leche Humana del estado de Maranhão presentan una alfabetización funcional en salud inadecuada. Los datos demuestran una asociación significativa entre el nivel de AS y factores como la edad, la escolaridad, los ingresos familiares y el resultado del MEEM. Las mujeres lactantes más jóvenes, con menor escolaridad, ingresos más bajos y signos de pérdida cognitiva leve presentaron una mayor prevalencia de AS inadecuada.

Ante los hallazgos de este estudio, cabe destacar que la alfabetización en salud, como base conceptual para la promoción de la salud, se configura como una estrategia eficaz para el cuidado de la salud de las mujeres lactantes. Su adopción como directriz en las políticas públicas puede favorecer el empoderamiento, el autocuidado y la ampliación del acceso cualificado a los servicios de salud, contribuyendo a la mejora de los resultados relacionados con la lactancia materna y la salud maternoinfantil.

Aunque la prevalencia de AS inadecuada en este estudio es elevada, se sugiere la introducción de recursos que mejoren la comunicación entre los profesionales de la salud y las pacientes, especialmente en el caso de las mujeres lactantes con bajos ingresos y bajo nivel educativo. Las medidas educativas son fundamentales para que los profesionales adapten su lenguaje, ajusten las orientaciones y prescripciones y desarrollen instrumentos de comunicación que satisfagan las necesidades y habilidades de las mujeres lactantes con mayor riesgo de déficits en la AS.

Se sugiere la inclusión de la alfabetización en salud en la formación y capacitación permanente de los profesionales de la atención primaria, con el fin de mejorar la atención a las mujeres lactantes. En la práctica asistencial, es esencial adoptar estrategias de comunicación adecuadas al perfil sociocultural de las usuarias. Futuras investigaciones, preferiblemente multicéntricas y cualitativas, podrían ampliar la comprensión del tema en diferentes contextos regionales.

## DISPONIBILIDAD DE DATOS

Todos los conjuntos de datos que respaldan los resultados de este estudio se publicaron en el propio artículo.
